# Sex-Specific Differences in the Association of Metabolically Healthy Obesity With Hyperuricemia and a Network Perspective in Analyzing Factors Related to Hyperuricemia

**DOI:** 10.3389/fendo.2020.573452

**Published:** 2020-10-06

**Authors:** Simiao Tian, Yazhuo Liu, Ao Feng, Shulong Zhang

**Affiliations:** ^1^ Department of Cardiology, Affiliated Zhongshan Hospital of Dalian University, Dalian, China; ^2^ School of Mathematical Sciences, Dalian University of Technology, Dalian, China; ^3^ Department of Clinical Nutrition and Metabolism, Affiliated Zhongshan Hospital of Dalian University, Dalian, China

**Keywords:** ****metabolic health, obesity, hyperuricemia, Bayesian network (BN), sex-specific

## Abstract

**Background:**

Although obesity is a well-known risk factor for hyperuricemia, it remains unclear whether obese subjects with metabolically healthy status have a decreased the risk of hyperuricemia and whether sex modifies the association of metabolically healthy obesity (MHO) with hyperuricemia risk. We aimed to investigate the sex-specific association between MHO and other obesity phenotypes and hyperuricemia, and to use Bayesian networks to determine and visualize the interactions among hyperuricemia and its related factors.

**Methods:**

This study was conducted using data from the China Health and Nutrition Survey 2009. Hyperuricemia was defined as serum uric acid ≥ 420 μmol/L in men and ≥ 360 μmol/L in women according to the guidelines. Body mass index (BMI) was used to define normal weight, overweight, and obese status in subjects, and metabolic health state was defined by the Adult Treatment Panel (ATP)-III and Visceral Adiposity Index (VAI) criteria, respectively. Subjects were categorized into six phenotypes according to their metabolic health and BMI level status.

**Results:**

Of the 7,364 Chinese adult individuals included, the prevalence of hyperuricemia among MHO women was only 8.5% (95% CI 4.8 to 14.3%), but increased to 30.7% among MUO women, whereas the highest prevalence among men was found in the MUOW phenotype (39.4%, 95% CI 35.4 to 43.6%), compared to 15.4% for male subjects with MHO. After adjusting for confounders, the MHO phenotype was significantly associated with an increased risk of hyperuricemia compared with their MHNW counterparts in women (OR: 1.95, 95% CI: 1.02–3.74) whereas a significant association was not found in men (OR: 1.46, 95% CI: 0.8–2.68). A complex network structure was established by BNs and then used to find connections between hyperuricemia and its related factors, as well as their interrelationships. By using BN reasoning, the probability of having hyperuricemia was 0.076 among MHO men, while it reached 0.124 in MHO women.

**Conclusions:**

In conclusion, our results demonstrated that the MHO phenotype was significantly associated with the risk of hyperuricemia only in women, not in men. This sex-specific differences in the association may suggest a favorable condition of MHO for Chinese men with respect to hyperuricemia risk, meanwhile more attention should be paid to the increased risk of hyperuricemia among MHO women.

## Introduction

Hyperuricemia is now becoming one of the most important public health concerns in international communities because of its rapid increase and significant impact on various clinical complications (1–3). Recent epidemiological studies indicate that 8.4–25% of the general Chinese population is diagnosed with hyperuricemia (4), and its prevalence is also very high in Japan (5) and in Western populations (6, 7). Among the general US adult population, the estimated number with elevated serum uric acid (SUA) was 21.4% in 2007–2008 (8) and remained substantial, albeit unchanged, between 2007 and 2016 (9). Hyperuricemia is related not only to an increased risk of gout but to many diseases, such as hypertension (10), diabetes mellitus (11), cardiovascular diseases, and overall mortality (12), that affect all segments of the population. In previous studies, individuals with coronary heart disease had higher SUA concentrations than their healthy counterparts (13, 14), and elevated SUA was found to have an adverse impact on mortality in the general adult population, particularly in older (>50 years) women (15).

Metabolic syndrome (MetS) refers to a cluster of interrelated metabolic abnormalities such as increased blood pressure, hyperglycemia, central adiposity, insulin resistance, and dyslipidemia (16, 17). It is well-recognized that MetS is a major risk factor for diabetes, cardiovascular diseases, and premature mortality (18–21), and previous studies also suggest a strong association between MetS and hyperuricemia and elevated SUA (22, 23). Along with MetS, obesity is another well-known determinant of many diseases, cardiovascular diseases (CVD), and mortality (24–28), and its causal relationship with an increased risk of hyperuricemia has also been advocated by previous studies (29, 30). However, obese people may differ from disease risk due to age, sex, or metabolically healthy status. Recent research has introduced a novel obesity phenotype characterized by favorable cardiometabolic profiles while being obese, and showed a favorable condition of metabolically healthy obesity (MHO) in terms of the incidence of diabetes, and CVD morbidity and mortality compared to normal-weight individuals (31–33). From recent large-scale prospective studies, MHO individuals had significantly lower mortality risk, approximately 20% for all-cause and other causes, and 27% for cardiovascular, when compared with the metabolically healthy non-obese (MHNO) group (32). Therefore, it is important for clinical studies that assess these associations to simultaneously take metabolic health and obesity status into account.

Of note, there are clear sex-specific disparities regarding the prevalence of hyperuricemia and/or SUA concentration, owing to various factors. Potential explanations for sex differences in the SUA level include genetic predisposition related to susceptibility to DNA methylation, differences in immune response such as interleukin levels, hormonal influences, and environmental factors such as differential response to exposures (34). In contrast, the association between MHO and a variety of adverse health consequences is also reported to be different between men and women (29, 35, 36).

To date, as far as we know, no consistent conclusions have been established regarding whether there is a sex-specific effect on the potential benefits of MHO status in terms of hyperuricemia risk in the Chinese population, and whether the degree and direction of the relationship between various obesity phenotypes and hyperuricemia differ by sex; therefore, this study aimed to investigate potential sex-specific aspects of the association of metabolic health and obesity with the risk of hyperuricemia in a population-level study of Chinese adults.

## Materials and Methods

### Study Population

This study used data from the China Health and Nutrition Survey (CHNS), a representative sample of the Chinese population. This survey is a large-scale longitudinal, household-based ongoing survey, and aims to examine the effects of health and nutrition across a set of large Chinese provinces. The comprehensive details of the CHNS and its sampling procedures have been described elsewhere (37). In brief, the CHNS study used a multistage, random cluster sampling method to select nationally representative households from 9 of the 31 mainland provinces. Within each selected household, original and new household members were longitudinally examined. Fasting blood samples from participants aged ≥7 years were collected for the first time in 2009. We used the data of the 2009 CHNS wave for the present study. Subjects had participated in health interviews and examinations, including blood sampling. Of the 11,929 participants whose data were used in the 2009 CHNS, individuals under 18 years old and those with incomplete data of anthropometric measures and blood sampling were excluded from the present study. As a result, a total of 7,364 individuals (3,419 men and 3,945 women) were enrolled in the present study. The CHNS study was approved by the institutional review committees of the University of North Carolina at Chapel Hill, the National Institute of Nutrition and Food Safety, Chinese Centers for Disease Control and Prevention, and the China-Japan Friendship Hospital, Ministry of Health (R01-HD30880, DK056350, and R01-HD38700). All participants provided written informed consent for the survey. All methods were performed in accordance with the relevant guidelines and regulations.

### Data Collection (Anthropometric and Biochemical Measurements)

This survey involved self-administered and standardized questionnaires, physical examinations, and biochemical measurements. The standardized questionnaire was required to be completed by the participants in a face-to-face interview with well-trained personnel, and it was used to collect information on sociodemographic characteristics (age, sex, marital status, and education level), behavioral factors (smoking status, alcohol intake, and physical activity), and their medications and self-reported family histories. Smoking status (yes [current or former], no) and alcohol drinking (yes [current or former], no) were defined according to participants’ smoking and alcohol drinking history.

The physical examination followed standardized procedures and evaluated anthropometric measurements and BP measured by well-trained examiners. Body weight and height were measured with participants barefoot and in light clothing to the nearest 0.1 kg and 0.1 cm, respectively. Waist circumference (WC) was measured with an inelastic tape to the nearest 0.1 cm at a midpoint between the bottom of the rib cage and the top of the iliac crest, following exhalation. Hip circumference was measured over thin clothing at the point of the maximum circumference of the buttocks. Both circumferences were measured to the nearest 0.1 cm. Body mass index (BMI) was calculated as weight (kg) divided by the square of height (meters). Systolic and diastolic blood pressures (SBP and DBP) were measured using a standardized mercury sphygmomanometer on an individual’s right arm. Three consecutive measurements of BP were taken on the same arm after a 10 min seated rest, and the mean of the three measurements was used for our analysis.

After an overnight fast, fasting blood samples were collected in the morning from participants using a standardized process and then analyzed in a national central clinical laboratory in Beijing. Plasma and serum samples were then frozen, and stored at −86°C for later laboratory analysis. Serum levels of fasting plasma glucose (FPG), total cholesterol (TC), high-density lipoprotein-cholesterol concentrations (HDL-C), triglyceride (TG), and uric acid (UA) and other routine blood biochemical indices were measured by a biochemical autoanalyzer. Details of all laboratory analyses and measurements can be found elsewhere (37). Homeostasis model assessment of insulin resistance (HOMA-IR) was calculated by the formula: HOMA-IR = fasting insulin (micro-international units per milliliter) × FPG (millimoles per liter)/22.5.

### Definitions of Hyperuricemia (Outcome Variable)

In our present study, hyperuricemia was defined as SUA *≥* 420 μmol/L in men and *≥* 360 μmol/L in women according to the guidelines (38).

### Definitions of Obesity and Metabolic Health

Obesity status was defined by using BMI cut-off points of 24 and 28 kg/m², and these cut-off points are based on the criteria advocated for Chinese adults (39, 40). Therefore, all participants were divided into three BMI groups: normal weight (BMI 18.5–23.9 kg/m²), overweight (BMI 24.0–27.9 kg/m²), and obese (BMI ≥28.0 kg/m²).

We defined metabolically unhealthy status based on two published criteria: (1) the Adult Treatment Panel-III (ATP-III) definition of metabolic syndrome (16), and individuals with more than two of the following metabolic abnormalities: (i) hypertension (SBP/DBP ≥ 130/85 mmHg or use of antihypertensive drugs); (ii) hypertriglyceridemia (TG≥1.7 mmol/L or use of lipid-lowering drugs; (iii) hyperglycemia (FPG≥5.6 mmol/L or use of medications for diabetes); and (iv) reduced HDL-C (HDL-C <1.04 mmol/L for men and <1.3 mmol/L for women). The WC criterion was not used because of its collinearity with BMI. (2) The Visceral Adiposity Index (VAI), where individuals with a lower VAI value can be considered to have a metabolically healthy status (i.e. being “metabolically healthy” was defined as VAI <1.59, and “metabolically unhealthy” was defined as VAI ≥1.59) (41).

### Definitions of Metabolic Obesity Phenotypes

According to the cross-classification of BMI categories and metabolic health status, study participants were categorized into one of the six following phenotypes: metabolically healthy normal-weight (MHNW); metabolically healthy overweight (MHOW); metabolically healthy obese (MHO); metabolically unhealthy normal-weight (MUNW); metabolically unhealthy overweight (MUOW); and metabolically unhealthy obese (MUO).

### Bayesian Networks Modeling

A Bayesian network (BN) is a statistical model to describe probabilistic relationships and reasoning among a set of variables using a directed acyclic graph (DAG) and can be thought of as a fusion of incidence diagrams and Bayes’ theorem (42–44). A BN can be represented by a tuple *B* = (*G, X, P*), with *G* = (*V, A*) and a DAG defined by two sets: vertices or nodes (denoted by *V*) and arcs or directed edges (denoted by *A*), X = {*X_i_*, …,*X_n_*} a set of random variables and *P* a joint probability distribution. Specifically, the nodes are represented as circles representing the random variables and directed edges symbolize the relationships between the variables. Providing that there is an edge from node *X_i_* to node *X_j_*, *X_i_* is then referred to as the parent of *X_j_*, emphasizing a statistical dependence between the corresponding variables; similarly, *X_j_* is referred to as the child of *X_i_*. The distribution *P* can be written as the product of the local probability of each random variable, conditional on their parent variables in the graph *G*:

$$P(X)=P(X_{i},\ldots ,X_{n})=P(X_{1})P(X_{2}|X_{1})\ldots P(X_{n}|X_{1},X_{2},\ldots ,X_{n-1})=\prod_{i=1}^{n}P(X_{i}|Pa(X_{i}))$$

*Pa*(*X_i_*) are the parents of *X_i_* in BN, and *P*(*X*) reflects the properties of BN (42, 45).

The process of estimating such a model is called *learning*, which consists of *structure learning* and *parameter learning*. Learning methods for both the parameters and the graphical structure of a BN are readily available (45). Briefly, learning Bayesian networks consists of finding the network that best fits the dataset for a certain scoring function. There are two main classes of algorithms for learning BN structures: constraint-based and score-based algorithms. The constraint-based algorithms aim to establish a set of conditional independence statements holding for the data, and use this set to build a network with d-separation properties corresponding to the determined conditional independence properties (42, 46). On the other hand, the score-based algorithms involve searching over possible Bayesian network structures in an attempt to maximize a scoring function. Scoring functions are generally a variation of likelihood penalized to discourage overly complex network structures (45).

### Bayesian Network Implementation

BNs were constructed from the data by the following steps: (1) apply one of the most commonly used heuristic algorithms, Tabu (47), for graphical structure learning along with the Bayesian Information Criterion score (48); (2) examine the stabilities of arcs in the networks from averaging 300 bootstrapped networks, and estimate the strengths of arcs by averaging the probability of the arcs presenting in the bootstrapped networks (49); (3) determine the structure and directions of arcs between variables using the averaged network; (4) query the conditional probability distributions (Bayesian reasoning) in the final networks and (5) visualize the final networks (50).

### Layers of Variables

The directions between certain variables were restricted by using a layering approach (51). First, the variables MetS and obesity, two components of metabolic health status, were not allowed to be linked to each other. Second, the variables MetS, obesity, and sex were allowed to be directed to hyperuricemia, and this setting ensured that information was embedded on the direction of causality for the sex-specific effect. Third, the variable hyperuricemia was not permitted to be directed to the abovementioned variables, i.e., some of the directions between variables were blacklisted in the network modeling (50).

### Statistical Analyses

The characteristics of the study population were presented as the mean and SD for continuous variables, or as numbers and percentages for categorical variables. Comparisons of the two sex groups were performed using Student’s t-test, and chi-square tests for categorical variables. Multivariate logistic regression analysis was conducted to estimate the overall and sex-specific association of various obesity phenotypes and hyperuricemia, with odds ratios (ORs) and 95% confidence intervals (CIs) calculated. The ORs and their 95% CIs for the association were adjusted sequentially for age, sex, urban/rural residence, smoking status, alcohol status, and metabolic health-obesity phenotypes (model 1), and then further adjusted for white blood cell, total cholesterol, LDL-C, hs-CRP, and diabetes variables (model 2). As there is no universal definition of metabolic health status, a sensitivity analysis was performed by using a new criteria based on the VAI (41), and metabolically healthy status was defined as VAI <1.59. Subgroup analyses were then conducted after categorizing the subjects according to general obesity, metabolic syndrome component, and metabolic status by sex. Furthermore, to understand the overall relationships between obesity phenotypes, adjusted variables, and hyperuricemia, as well as the interrelationship between those adjusted variables, Bayesian networks were used for the analysis and especially for estimating conditional probabilities of having hyperuricemia given various adjusted variable values simultaneously. All statistical analyses were performed using R version 3.2.2 software (52), and a *P*-value <0.05 was considered statistically significant. The *bnlearn* package (50) of the R environment was used to conduct the whole analysis of BN modeling, including network structure learning, parameter estimation, the stabilities and strengths of network arcs, and query of the conditional probability distributions in the finalized network and visualization.

## Results

### Characteristics of the Sample Population

**Figure 1:**
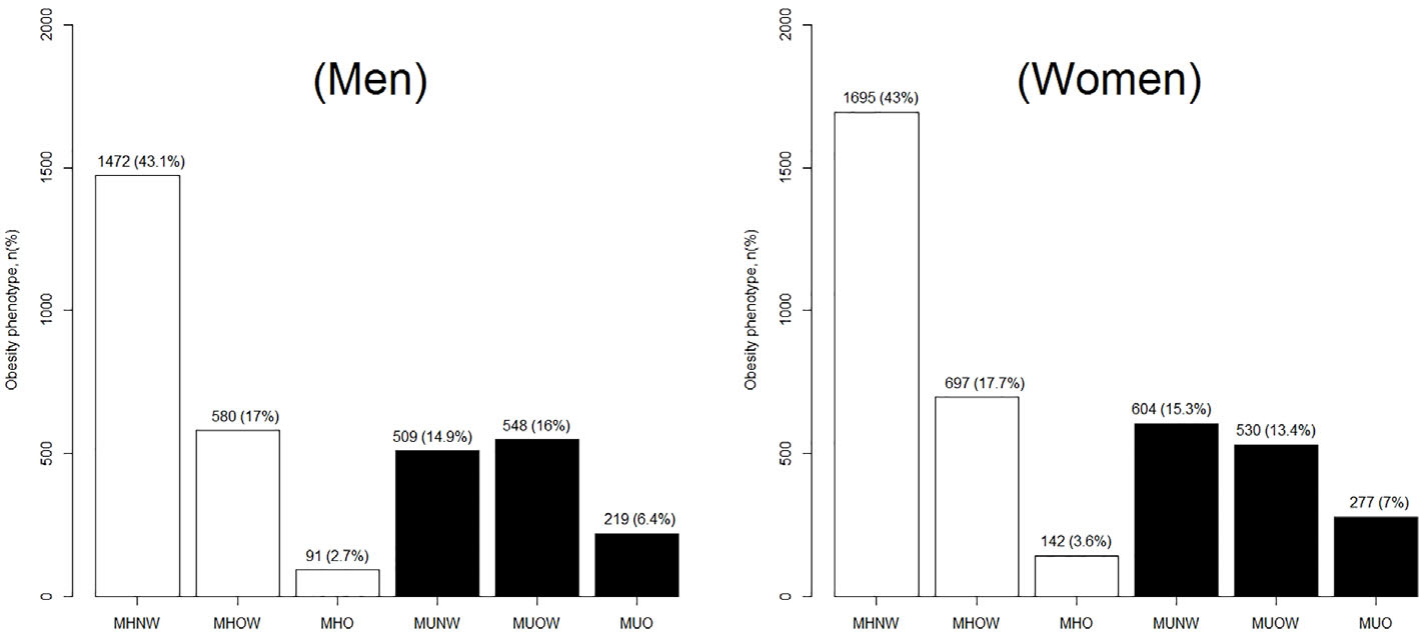
Distribution of ATP-III criteria-based metabolic obesity phenotypes according to by sex. ATP-III, the Adult Treatment Panel-III; MHNW, metabolically healthy normal-weight; MHOW, metabolically healthy overweight; MHO, metabolically healthy obese; MUNO, metabolically unhealthy non-obese; MHO, metabolically healthy obese; MUNW, metabolically unhealthy normal-weight; MUOW, metabolically unhealthy overweight; and MUO, metabolically unhealthy obese.

A total of 7,364 adult participants with a mean age of 50.30 ± 14.45 years were included in this analysis. The general and sex-specific characteristics of the study population are presented in **Table 1**. A total of 3,945 participants (53.6%) were female, which reflects an almost even distribution of men and women. Smoking and alcohol intake were more prevalent among men than women (smoking: 62 *vs.* 3.8%; alcohol intake: 61.1 *vs.* 8.7%, respectively). The occurrence of general obesity (including overweight) was similar between men and women (42.06 *vs.* 41.72%), whereas abdominal obesity was more common among women than men (56.9 *vs.* 49.6%). **Figure 1** showed ATP-III criteria-based metabolic obesity phenotypes according to sex, the percentages of individuals classified as MHNW (43.05 *vs.* 42.97%), MHOW (16.96 *vs.* 17.67%), MUNW (14.89 *vs.* 15.31%) and MUO (6.41 *vs.* 7.02%) were similar in men and women, respectively. However, a higher proportion of women were classified as MHO (2.66 *vs.* 3.6%), and a higher proportion of men were classified as MUOW (16.03 *vs.* 13.43%), when compared to their opposite sex counterparts (**Figure 1**).

**Table 1:** Study sample characteristics of subjects according to sex.

	Total	Men	Women	*P*-value
N	7,364	3,419	3,945	
Age, years	50.30 ± 14.45	50.31 ± 14.63	50.29 ± 14.3	0.972
Weight, kg	61.63 ± 10.58	66.09 ± 10.57	57.77 ± 8.95	<0.001
Height, cm	161.08 ± 8.53	167.04 ± 6.6	155.91 ± 6.37	<0.001
BMI, kg/m^2^	23.68 ± 3.15	23.62 ± 3.11	23.73 ± 3.19	0.156
Waist circumference, cm	83.31 ± 9.76	84.95 ± 9.58	81.89 ± 9.68	<0.001
Hip circumference, cm	95.01 ± 7.49	95.21 ± 7.33	94.85 ± 7.62	0.038
HDL-C, mmol/L	1.43 ± 0.46	1.38 ± 0.46	1.47 ± 0.45	<0.001
LDL-C, mmol/L	3.00 ± 0.98	2.94 ± 0.97	3.05 ± 0.99	<0.001
DBP, mm Hg	80.73 ± 11.26	82.35 ± 10.97	79.33 ± 11.33	<0.001
SBP, mm Hg	124.76 ± 18.73	126.11 ± 17.25	123.6 ± 19.84	<0.001
FPG, mmol/L	5.32 ± 1.24	5.38 ± 1.4	5.27 ± 1.09	<0.001
TC, mmol/L	4.88 ± 1.00	4.83 ± 0.97	4.92 ± 1.03	<0.001
TG, mmol/L	1.68 ± 1.46	1.8 ± 1.67	1.58 ± 1.23	<0.001
Urea, mmol/L	5.46 ± 1.55	5.8 ± 1.56	5.16 ± 1.48	<0.001
Uric Acid, μmol/L	307.95 ± 105.42	356.14 ± 111.38	266.18 ± 78.98	<0.001
HOMA-IR	3.65 ± 6.74	3.76 ± 6.96	3.55 ± 6.55	0.171
hsCRP	2.42 ± 6.35	2.67 ± 7.5	2.21 ± 5.15	0.002
HbA1c	5.58 ± 0.75	5.59 ± 0.8	5.57 ± 0.72	0.216
Smoker, n (%)	2,271 (30.8%)	2,121 (62%)	150 (3.8%)	<0.001
Alcohol drinker, n (%)	2,434 (33.1%)	2,090 (61.1%)	344 (8.7%)	<0.001
Urban resident, n (%)	5,033 (68.3%)	2,347 (68.6%)	2,686 (68.1%)	0.624
Diabetes, n (%)	378 (5.1%)	210 (6.1%)	168 (4.3%)	<0.001
Dyslipidemia, n (%)	2,548 (34.6%)	1,286 (37.6%)	1,262 (32%)	<0.001
Components of ATP-III- based MetS criteria				
Reduced HDL-C, n (%)	1,898 (25.8%)	605 (17.7%)	1,293 (32.8%)	<0.001
Elevated BP, n (%)	3,060 (41.6%)	1,576 (46.1%)	1,484 (37.6%)	<0.001
Elevated TG, n (%)	2,406 (32.7%)	1,226 (35.9%)	1,180 (29.9%)	<0.001
Elevated FPG, n (%)	1,805 (24.5%)	896 (26.2%)	909 (23%)	0.002
Abdominal obesity, n (%)	3,942 (53.5%)	1,697 (49.6%)	2,245 (56.9%)	<0.001
MetS, n (%)	2,687 (36.5%)	1,276 (37.3%)	1,411 (35.8%)	0.175
Age groups				
18–39 years	1,834 (24.9%)	873 (25.5%)	961 (24.4%)	0.183
40–59 years	3,645 (49.5%)	1,653 (48.3%)	1,992 (50.5%)	
≥60 years	1,885 (25.6%)	893 (26.1%)	992 (25.1%)	
BMI levels				
Normal weight, n (%)	4,280 (58.1%)	1,981 (57.9%)	2,299 (58.3%)	0.038
Overweight, n (%)	2,355 (32.0%)	1,128 (33.0%)	1,227 (31.1%)	
Obese, n (%)	729 (9.9%)	310 (9.1%)	419 (10.6%)	

Data are reported as the mean (SD) or n (%).

BMI, body mass index; HDL-C, high-density lipoprotein cholesterol; LDL-C, low-density lipoprotein cholesterol; DBP, diastolic blood pressure; SBP, systolic blood pressure; FPG, fasting plasma glucose; TC, total cholesterol; TG, triglycerides; HOMA-IR, homoeostatic model assessment of insulin resistance; hsCRP, high-sensitivity C-reactive protein; ATP-III, the Adult Treatment Panel-III; MetS, metabolic syndrome.

### Prevalence of Hyperuricemia by Sex

The prevalence of hyperuricemia, according to the categories of metabolic health status and BMI levels is shown in **Table 2**. In women, the prevalence increased with weight for both metabolically healthy and metabolically unhealthy subjects. The prevalence of hyperuricemia among obese females with favorable metabolic profiles (MHO) was only 8.5% (95% CI 4.8 to 14.3%), but increased to 18.7% (95% CI 15.8 to 22%) when normal-weight subjects fulfilled a metabolically unhealthy status (MUNW) and further increased up to 30.7% among MUO subjects (**Table 2**). However, in men, overweight subjects consistently revealed a higher prevalence than their obese counterparts regardless of metabolic health status. The highest prevalence was found in the MUOW phenotype and nearly 40% of MUOW subjects suffered from hyperuricemia (39.4%, 95% CI 35.4 to 43.6%), compared to 38.4% of those obese males who were metabolically unhealthy. Additionally, for male subjects with MHO, the prevalence of hyperuricemia was 15.4%, slightly lower than their overweight counterparts (15.4 *vs.* 16.6%) (**Table 2**).

**Table 2:** Sex-specific prevalence of hyperuricemia according to metabolic health and obesity status (metabolic obesity phenotypes).

Metabolic health status	BMI levels	Men	Women
Metabolically healthy	Normal weight (MHNW)	11.4 (9.9–13.1)	4 (3.2–5.1)
	Overweight (MHOW)	16.6 (13.7–19.8)	5.5 (4–7.4)
	Obese (MHO)	15.4 (9.3–24.3)	8.5 (4.8–14.3)
Metabolically unhealthy	Normal weight (MUNW)	23.6 (20.1–27.5)	18.7 (15.8–22)
	Overweight (MUOW)	39.4 (35.4–43.6)	23 (19.6–26.8)
	Obese (MUO)	38.4 (32.2–44.9)	30.7 (25.5–36.4)

The metabolic health status was defined by ATP-III; the obesity status was defined by body mass index. Abbreviations: ATP-III, the Adult Treatment Panel-III; MHNW, metabolically healthy normal-weight; MHOW, metabolically healthy overweight; MHO, metabolically healthy obese; MUNO, metabolically unhealthy non-obese; MHO, metabolically healthy obese; MUNW, metabolically unhealthy normal-weight; MUOW, metabolically unhealthy overweight; and MUO, metabolically unhealthy obese.

In addition, the sex-specific prevalence of hyperuricemia, based on VAI criteria-based metabolic health status are shown in **Supplementary Figure 1**. Generally, a similar pattern of prevalence was noted in both men and women, and the prevalence of hyperuricemia was 12.1 (95% CI 6.7 to 20.5%) in MHO men and 9.1% (95% CI 4.8 to 16.1%) in MHO women (**Supplementary Figure 1**).

### Overall and Sex-Specific Associations Between MHO and Hyperuricemia

The overall and sex-specific associations of different obesity phenotypes with hyperuricemia are presented in **Table 3**. Of the total subjects, the age-adjusted ORs (95% CI) for hyperuricemia were 1.6 (95% CI: 1.04–2.47) for MHO subjects, 3.22 (95% CI: 2.63–3.93) for MUNW, and 6.56 (95% CI: 5.2–8.28) for MUO when compared to those with MHNW (model 1), which demonstrates a strong association between hyperuricemia and any obesity phenotype. This independent association persisted significantly even after further adjustment for LDL-C, hs-CRP, and other biomarkers (model 2). The association for hyperuricemia was significant but consistently attenuated for metabolically unhealthy subjects regardless of BMI levels, with corresponding ORs of 2.37 (95% CI: 1.92–2.93), 3.92 (95% CI: 3.21–4.78), and 4.69 (95% CI: 3.66–6.01) for MUNW, MUOW, and MUO subjects, respectively. Moreover, those with MHO were still significantly associated with a higher risk of hyperuricemia and the degree of association was even stronger in model 2 (OR = 1.68, 95% CI 1.08–2.6).

**Table 3:** Odds ratios and 95% confidence intervals for the association between MHO and hyperuricemia by sex.

ATP-III-based criteria	Total (OR, 95% CI)		Men (OR, 95% CI)		Women (OR, 95% CI)	
	Model 1	Model 2	Model 1	Model 2	Model 1	Model 2
MHNW	1 (Reference)	1 (Reference)	1 (Reference)	1 (Reference)	1 (Reference)	1 (Reference)
MHOW	1.44 (1.15–1.8)	1.49 (1.18–1.87)	1.51 (1.15–1.99)	1.63 (1.23–2.15)	1.27 (0.84–1.91)	1.28 (0.84–1.93)
MHO	**1.6** **(1.04–2.47)**	**1.68 (1.08–2.6)**	1.26 (0.69–2.28)	1.46 (0.8–2.68)	**2.02 (1.06**–**3.85)**	**1.95 (1.02**–**3.74)**
MUNW	3.22 (2.63–3.93)	2.37 (1.92–2.93)	2.48 (1.91–3.23)	1.74 (1.31–2.32)	4.24 (3.06–5.88)	3.34 (2.39–4.69)
MUOW	5.43 (4.49–6.55)	3.92 (3.21–4.78)	5.1 (4.02–6.46)	3.57 (2.77–4.6)	5.68 (4.12–7.84)	4.45 (3.2–6.2)
MUO	6.56 (5.2–8.28)	4.69 (3.66–6.01)	4.73 (3.44–6.5)	3.47 (2.46–4.91)	8.91 (6.23–12.74)	6.31 (4.33–9.18)

Data are presented as odds ratios (95% confidence intervals).

ATP-III, the Adult Treatment Panel-III; MHNW, metabolically healthy normal-weight; MHOW, metabolically healthy overweight; MHO, metabolically healthy obese; MUNO, metabolically unhealthy non-obese; MHO, metabolically healthy obese; MUNW, metabolically unhealthy normal-weight; MUOW, metabolically unhealthy overweight; and MUO, metabolically unhealthy obese. Model 1: Adjusted for age, urban/rural resident, smoking status, alcohol status, and metabolic health-obesity phenotypes. Model 2: Adjusted for Model 1+ white blood cell, total cholesterol, LDL-C, hsCRP and diabetes.

The bold values mean the results are significant for MHO groups.

In men, the age-adjusted OR for having hyperuricemia in MHO subjects was 1.26 (95% CI: 0.69–2.28), indicating that those subjects were unlikely to have a greater risk of hyperuricemia compared to their MHNW peers. This association was still non-significant when adjusting for various biomarkers (model 2), even though the magnitude of association raised to 1.46 (95% CI: 0.8–2.68) (**Table 3**). However, compared to the MHNW phenotype, subjects in other obesity phenotypes were consistently associated with an increased risk of hyperuricemia; the corresponding ORs ranged from 1.51 in MHOW to 5.10 in MUOW and from 1.63 to 3.47 based on age- and fully adjusted models, respectively. In contrast, female subjects with MHO were nearly 2-fold more likely to have hyperuricemia than their MHNW peers, with an aged-adjusted OR of 2.02 (95% 1.06–3.85). A similar pattern between MHO and hyperuricemia was found after further accounting for confounders (model 2): the positive association was relatively attenuated but still significant, and the OR was 1.95 (95% CI: 1.02–3.74) when compared with female MHNW subjects. Furthermore, female subjects with any unfavorable BMI and metabolic health profiles were at a higher risk of having hyperuricemia when compared to their normal-weight peers with metabolically healthy status (**Table 3**). Notably, the risk was substantially amplified in the female MUO phenotype, which increased up to an 8.91- and 6.31-fold risk of hyperuricemia in the age- and fully adjusted models, respectively.

Furthermore, when using VAI criteria-based metabolic health status (the sensitivity analysis), a highly similar pattern was found: the association between MHO and hyperuricemia was only significant in women, not in men, which justified a sex-specific effect on this association (**Supplementary Table 1**). To assure the reliability of this sex-specific difference, a multivariate logistic regression was accompanied by stepwise variable selection. This sensitivity analysis revealed a highly similar pattern that only MHO women were significantly associated with hyperuricemia when compared to their MHNW peers, but not in MHO men (**Supplementary Table 2**).

### Sex-Specific Association Between BMI, ATP-III Components and Metabolic Status and Hyperuricemia

**Figure 2:**
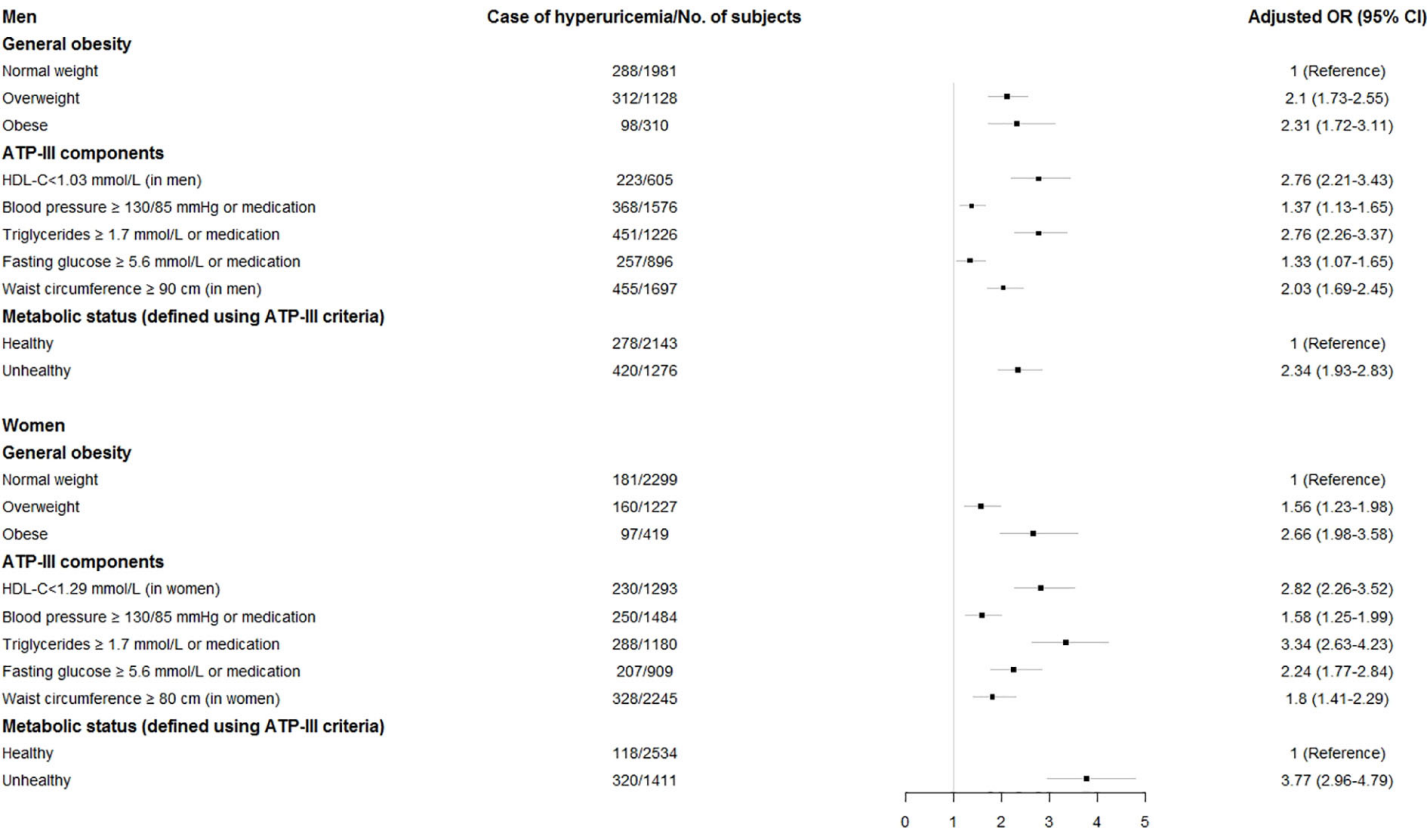
Adjusted odds ratios (OR) and 95% confidence intervals (CI) for hyperuricemia associated with Adult Treatment Panel-III metabolic components, body mass index categories, and metabolic status by sex. Horizontal bars are 95% CIs. The adjusted OR was obtained from model 2 which was adjusted for age, urban/rural resident, smoking status, alcohol status, and metabolic health-obesity phenotypes, white blood cell, total cholesterol, LDL-C, hs-CRP, and diabetes.

Subgroup analyses were conducted to separately present the sex-specific associations of BMI categories, ATP-III components and metabolic status with hyperuricemia risk, and the results are shown in **Figure 2**, with results for men at the top and those for women at the bottom. In men, compared with normal-weight subjects, both overweight and obese subjects were at an increased risk of hyperuricemia, with adjusted ORs of 2.1 (95% CI 1.73–2.55) and 2.31 (95% CI 1.72–3.11), respectively. The positive association with risk of hyperuricemia was significant for all components of the ATP-III criteria, consistently showing a greater than two-fold risk for all components except blood pressure, which did reveal a relatively lower association (OR: 1.37, 95% CI 1.13–1.65). Metabolically unhealthy subjects (irrespective of obesity) were at a higher risk of developing hyperuricemia (OR = 2.34, 95% CI 1.93–2.83) than their metabolically healthy counterparts (**Figure 2**, top).

In women, a similar pattern was noted in which these associations were still significant, albeit for most of cases, the risk of having hyperuricemia was higher when compared to men (**Figure 2**, bottom). Similarly, women with overweight (OR: 1.56, 95% CI 1.23–1.98) and obesity (OR: 2.66, 95% CI 1.98–3.58) also had an increased risk of hyperuricemia compared with their counterparts with a healthy BMI. Among all components of the ATP-III criteria, elevated TG was most strongly associated with hyperuricemia (OR = 3.34, 95% CI 2.63–4.23). Moreover, the adjusted ORs associated with metabolically unhealthy women for hyperuricemia were consistently significant, with a 3.77-fold increased risk compared with metabolically healthy women.

### Bayesian Network Model

**Figure 3:**
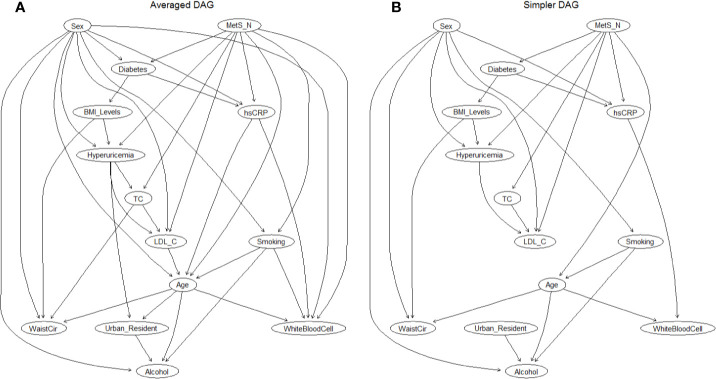
The DAG underlying the Bayesian network learned from the covariates and hyperuricemia. **(A)** Averaged DAG with strength of arcs greater than 0.5; **(B)** Simplified DAG derived from the averaged DAG after retaining arcs with a strength greater than 0.85. Abbreviations: DAG, directed acyclic graph; MetS, metabolic syndrome; TC, total cholesterol; LDL-C, low-density lipoprotein cholesterol; WaistCir, waist circumference.

After demonstrating a sex-specific differences in the association between MHO and hyperuricemia, we further extended the investigation to understand how changes in metabolic health status, BMI levels, and other risk factors can affect the risk of hyperuricemia, as well as to simultaneously explore interrelationships among hyperuricemia and their related risk factors. Logistic regression modeling can only be used to evaluate the associated risk intensity between obesity phenotypes and hyperuricemia, but did not enable to reflect the complex relationship between metabolic health, BMI levels, risk factor, and outcomes because it required independence among variables; therefore, a BN modeling was finally used for this special purpose, as well as for estimating the conditional probability rather than selecting the significant variables (**Figure 3**). By learning 300 networks from the data, an averaged BN was constructed with arcs appearing at least in 50% of bootstrapped networks, and is shown in **Figure 3A**. This BN model described complex relationships between various factors and hyperuricemia, as well as the relationship among risk factors and demographic covariates. All the directions of the arcs appear to be well established: this could be due to the use of a *Whitelist* and a *Blacklist* (the layering approach) since they restrict the directions of arcs.

Then, a further examination of the arc strengths was performed by using a threshold of 0.85, and the resulting BN confirmed that 25 out of 37 arcs in the network appear with a frequency of at least 0.85. **Figure 3B** showed this simplified BN consisting of 14 nodes and 25 directed arcs while losing little information in the process (the direct comparison of arcs between the averaged BN and simplified BN is shown in **Supplementary Figure 2**). Directed edges represent probabilistic dependencies between the nodes that are connected rather than the causal relationship between hyperuricemia, demographic covariates, and potential risk factors. The connections between hyperlipidemia and its related factors were established by a complex network structure, in which a direct connection between metabolic syndrome, sex, BMI levels, LDL-C, and hyperuricemia was found (**Figure 3B**), as well as an indirect link between diabetes and hyperuricemia through BMI levels. In addition, MetS and sex were directly connected with six covariates, indicating that they had the most children nodes and implicitly the sex-specific relationship. The interrelationship between the related factors of hyperuricemia is also exhibited in **Figure 3B**. For instance, smoking, alcohol intake, and waist circumference were related to sex and age; hs-CRP was associated with MetS and sex, and abdominal obesity (WC) was also associated with body mass (BMI levels).

### Reasoning Model

We can also use BNs to predict the conditional probability of having a hyperuricemia event given various evidence from specific nodes. **Table 4** summarizes the conditional probability of having hyperuricemia in the BMI group and the number of metabolic diseases. The conditional probability of having hyperuricemia with a normal weight and metabolically healthy status was only 0.05, whereas the probability doubled (probability 0.1) with obesity. When the number of metabolic diseases accumulated up to four, the probability increased to 0.56. Next, when taking into account sex, the MHO men had a 0.076 chance of occurrence of hyperuricemia, while this chance reached 0.124 in MHO women. When three concomitants of obesity, metabolic unhealthy status, and diabetes were present, the probability increased to 0.606.

**Table 4:** The conditional probability distribution table of hyperuricemia events given different metabolic health and obesity statuses, sexes, and the presence of diabetes.

BMI group	Number of metabolic components	Other factors	Hyperuricemia Probability
Normal weight	0		0.05
Obese	0		0.10
Obese	1		0.14
Obese	2		0.31
Obese	4		0.56
Obese	0	Sex: Men	0.076
Obese	0	Sex: Women	0.124
Obese	4	Presence of Diabetes: Yes	0.606

## Discussion

In this nationally representative cross-sectional study using Chinese adults, we examined the association between various obesity phenotypes and hyperuricemia, with a specific focus on sex-specific effects. As one major finding, a significantly positive association between MHO and hyperuricemia was found in women only, after adjusting for potential confounders, whereas metabolically unhealthy status was associated with hyperuricemia regardless of sex. To the best of our knowledge, this is the first large and population-based study that investigated the sex-specific relationship between MHO and hyperuricemia in a Chinese population.

Several prospective and observational studies have reported the relationship between obesity, metabolic syndrome, and the risk of hyperuricemia (22, 23, 53), but scarce have focused on sex-specific differences in this association, especially in metabolically healthy subjects. A recent study including 5,591 Korean adults from Kim and colleagues (29) observed that women with concomitant MetS and obesity carried a nearly 2.5-fold higher risk of hyperuricemia compared to men with both conditions (OR: 7.24 *vs.* 2.90). Intriguingly, although MHO in men was significantly associated with hyperuricemia, women with the MHO phenotype still had a higher risk than men, with OR values of 4.6 and 1.6, respectively. In addition, the association of male MHO was just at the borderline of significance (29). From a large study of 310,577 middle-aged Japanese adults undergoing periodic health examinations (53), Shirasawa et al. found that obese men and women without central obesity were associated with 1.33- and 3.76-fold higher odds of hyperuricemia risk compared with their normal-weight counterparts, respectively, and more importantly, a higher risk was still found in obese women. Of note, in Shirasawa et al.’s study, even though the MHO phenotype was not explicitly defined by using any of the MetS criteria as in the present study, obesity without central obesity can be roughly considered a surrogate of the MHO phenotype. It is also noteworthy that both of the abovementioned studies focused on the worst status of obesity and metabolic health, which were the MUO phenotype (29) and obesity with central obesity (53), respectively. Although there is some discrepancy in the way that obesity and metabolic health status were defined, our results were in agreement with previous studies and support a sex-specific difference in the association of MHO with hyperuricemia, that is, only MHO women were at a significant 2-fold higher risk of hyperuricemia compared to their MHNW peers; this association persisted after adjustment for potential confounding factors. Another small sample study, performed by Elizalde-Barrera et al. among 88 nondiabetic Mexican patients free of cardiovascular disease, showed a significant correlation between HOMA-IR and SUA in women, especially in obese women, but not in men in general (54). This also supports our evidence of a sex-specific effect because the metabolic health status can be defined by the HOMA-IR criterion (55), and more specifically, only obese women with higher HOMA-IR, a surrogate of the MHO phenotype, were significantly positively related to elevated SUA levels. Combining the findings from the present study and above, it seems reasonable to suggest that the association between the MHO phenotype and hyperuricemia is more prominent in females than in males. Therefore, more attention should be paid to the female population with metabolically healthy status in the clinical guidelines.

Although the link between obesity and elevated SUA/hyperuricemia is more robust among metabolically healthy women than in men, the mechanism underlying this sex-specific association remains unclear. However, several studies have suggested a role for estrogen and/or sex hormone levels in this particular relationship. It is well known that estrogen is an uricosuric agent and thereby may promote the excretion of uric acid, which in turn leads to this sex difference (56). An early study, performed by Anton et al. found a higher renal clearance of urate in women and argued that this was due to their higher plasma estrogen levels (57). From a study of 7,662 American women aged 20 years and older, it was found that menopause was independently related to higher SUA levels (58), which may underline a greater magnitude of association among MHO women in special age intervals. Another small study on 128 obese patients who underwent laparoscopic sleeve gastrectomy showed that increased estradiol levels and estradiol/total testosterone ratios in obese female patients might be related to SUA improvement (59). Moreover, the evidence also indicated a potential impact of estrogen on fat metabolism and fat distribution in women. For example, Zhang et al. demonstrated that fat accumulation in the abdominal cavity was more pronounced as estrogen levels in postmenopausal women decreased, which led to abdominal obesity (59). Future investigations are warranted to explore the underlying mechanisms involved in the sex-specific association between the MHO phenotype and hyperuricemia risk.

Until now, investigating the association of MetS and its components with hyperuricemia is still an ongoing field of research (23, 60, 61), although there are discrepancies as to which MetS components play a pivotal role in this association. In a case-control study of 193 Mediterranean subjects, Vaya et al. found that subjects with MetS had a nearly 3-fold increased risk of hyperuricemia compared with their counterparts without MetS (23). Low HDL-C and high-glucose were only two independent predictors for hyperuricemia among all the MetS components, with adjusted ORs of 2.71 and 2.14, respectively. A recent Japanese cohort study also found a significant association of both baseline and changes in SUA with incident MetS; the significant association was more pronounced between SUA and obesity and high triglycerides (61). The finding of a significant association between SUA and the MetS component of obesity was also confirmed by previous studies (60, 62, 63), and more importantly, the increased risk was more pronounced in overweight and obese women (60, 64), and that the magnitude of SUA association with the development of MetS was greater in women than in men (22). Our results were in line with these previous findings and showed a consistent positive association between MetS and the risk of hyperuricemia in both men and women, irrespective of BMI levels. Moreover, a greater magnitude of association in women than men was observed among all except the WC component. Moreover, the results of this study were similar and consistent with other large epidemiological studies that have observed a positive relationship of lower HDL-C (65, 66), elevated BP (65), TG (66, 67), FPG (68), and abdominal obesity (69) with hyperuricemia. Of note, a recent meta-analysis confirmed a significant dose-response relationship between the SUA levels and the incidence of MetS, with an augmentation of 5% risk of MetS for each 1 mg/dl increase in SUA, and more importantly, a significant sex-specific difference in the association was found, with a higher risk in females than males (70).

BN modeling enables simultaneous exploration of interrelationships among a large number of diseases and their related risk factors, without affecting the interpretability of the network (43, 49). Another advantage of BN modeling is that, thanks to Markov blanket theory, complex models can be divided into a collection of simpler models that which are mathematically tractable and computationally simpler (71). For instance, in the present study, the Bayesian network model displays connections between hyperuricemia and its related risk factors that were established by a complex network structure, along with easy interpretability of the network. Of these, direct connections were found between metabolic syndrome, sex, BMI levels, LDL-C, and hyperuricemia (**Figure 3**) whereas diabetes was indirectly connected to hyperuricemia *via* BMI levels. Moreover, BN modeling can be used as high-level abstraction for reflecting and investigating hypotheses on whether and how risk factors are related to each other, whereas traditional logistic regression suffers from its limitations of independency (43, 72). For instance, age was related to waist circumference and smoking and drinking status, and metabolic syndrome was directly connected with diabetes and hs-CRP. Therefore, the BN model can be applied to simultaneously explore the dependency of hyperuricemia on all risk factors, and the interrelationships between these factors in the model.

Furthermore, the BN model can always be used for qualitative and quantitative reasoning in the context of probability inferences of an unknown node (hyperuricemia) based on the built network (43, 45). For example, according to our reasoning model (**Figure 3B**), when a metabolically healthy normal-weight subject became obese, the probability of having hyperuricemia increased from 5 to 10% whereas this probability was up to 31% if this subject remained obese and had any two components of MetS. If a metabolically healthy male subject was obese, then he had a 7.6% probability of having hyperuricemia, but the probability increased to 12.4% when this subject was female, which emphasizes a remarkable sex-specific difference. Diabetes is indirectly related to hyperuricemia: if the MUO subject had diabetes, the probability of hyperuricemia exceeded 60%. Therefore, under the advantage of flexible reasoning mechanism and easy interpretability, BN models can be used as an efficient method for disease identification and diagnosis.

Several limitations of this study should be acknowledged. First, due to the nature of the cross-sectional design, we can only identify the association and its magnitude between MHO and hyperuricemia risk, and causality cannot be determined, even using BN modeling; further cohort studies are warranted to clarify our findings. In addition, the studied population included only Chinese subjects, so generalizability of the results to a wider population should be undertaken with caution. Nevertheless, the notable strengths and contributions of this study should be mentioned. First, the large sample size of our study guaranteed a reasonable statistical power that likely reflects real sex-specific associations. Second, our study offers a potential compelling application of BN in general medicine: BN not only provides an intuitive qualitative description of the relationship between hyperuricemia and its relevant factors, but also provides quantitative reasoning that can be used to explore the interrelationships among these factors and test novel hypotheses by simulation.

## Conclusion

In conclusion, by using a nationwide, population-based Chinese cohort, our study reports compelling evidence that the MHO phenotype was significantly associated with the risk of hyperuricemia, and more importantly, the association revealed sex differences that were only found in MHO women, not in men, which suggests a favorable condition of MHO for Chinese men with respect to hyperuricemia risk. In addition, we applied BN modeling to show the interrelationships between hyperuricemia and its related factors. BN modeling provides insights that will help to identify appropriate target populations and relevant risk factors for managing hyperuricemia risk.

## Data Availability Statement

Publicly available datasets were analyzed in this study. This data can be found here: http://www.cpc.unc.edu/projects/china/data.

## Ethics Statement

The studies involving human participants were reviewed and approved by the University of North Carolina at Chapel Hill, the National Institute of Nutrition and Food Safety, Chinese Centers for Disease Control and Prevention, and the China-Japan Friendship Hospital. The patients/participants provided their written informed consent to participate in this study.

## Author Contributions

Conceptualization: SZ and ST. Data acquisition: YL and AF. Statistical Analysis: ST. Investigation: SL and YL. Writing—original draft: ST and ZL. Writing—review and editing: SZ, YL, and AF. All authors contributed to the article and approved the submitted version.

## Funding

This research was funded by National Natural Science Foundation of China (81803329) and China Postdoctoral Science Foundation (2018M631780).

## Conflict of Interest

The authors declare that the research was conducted in the absence of any commercial or financial relationships that could be construed as a potential conflict of interest.
