# Supplementary material for: Sex-Specific Differences in the Association of Metabolically Healthy Obesity With Hyperuricemia and a Network Perspective in Analyzing Factors Related to Hyperuricemia
Source: Front Endocrinol (Lausanne). 2020 Oct 6;11:573452. doi: 10.3389/fendo.2020.573452 (PMC7573308; doi:10.3389/fendo.2020.573452)

Supplementary Material

**Supplementary Table 1**. Odds ratios and 95% confidence intervals for the association between VAI-based metabolic health and obesity phenotypes and hyperurcemia by sex. Data are presented as odds ratios (95% confidence intervals).

|  | Total (OR, 95% CI) | | Men (OR, 95% CI) | | Women (OR, 95% CI) | |
| --- | --- | --- | --- | --- | --- | --- |
| VAI -based criteria | Model 1 | Model 2 | Model 1 | Model 2 | Model 1 | Model 2 |
| MHNW | 1(Reference) | 1 (Reference) | 1(Reference) | 1 (Reference) | 1(Reference) | 1 (Reference) |
| MHOW | 1.67 (1.3-2.15) | 1.79 (1.39-2.32) | 1.78 (1.33-2.39) | 1.98 (1.47-2.67) | 1.4 (0.84-2.33) | 1.41 (0.84-2.37) |
| MHO | **1.68 (1.04-2.73)** | **1.82 (1.12-2.97)** | 1.19 (0.62-2.3) | 1.44 (0.74-2.81) | **2.4 (1.17-4.93)** | **2.31 (1.11-4.82)** |
| MUNW | 4.03 (3.29-4.93) | 2.97 (2.4-3.67) | 3.73 (2.88-4.83) | 2.67 (2.04-3.51) | 3.98 (2.81-5.63) | 3.16 (2.22-4.51) |
| MUOW | 5.7 (4.68-6.94) | 4.11 (3.35-5.04) | 5.88 (4.61-7.5) | 4.1 (3.16-5.31) | 4.89 (3.46-6.91) | 3.92 (2.75-5.57) |
| MUO | 8.12 (6.37-10.34) | 5.56 (4.3-7.18) | 6.21 (4.48-8.6) | 4.41 (3.11-6.26) | 9.42 (6.41-13.85) | 6.48 (4.34-9.69) |

*Abbreviations*: VAI, visceral adiposity index; MHNW, metabolically healthy normal-weight; MHOW, metabolically healthy overweight; MHO, metabolically healthy obese; MUNO, metabolically unhealthy non-obese; MHO, metabolically healthy obese; MUNW, metabolically unhealthy normal-weight; MUOW, metabolically unhealthy overweight; and MUO, metabolically unhealthy obese. Model 1: Adjusted for age, urban/rural resident, smoking status, alcohol status and metabolic health-obesity phenotypes. Model 2: Adjusted for Model 1+ white blood cell, total cholesterol, LDL-C, hsCRP and diabetes.

**Supplementary Table 2**. Odds ratios and 95% confidence intervals for the association between each adjusted variable/predictor variable and and hyperurcemia by sex based on the fully model and stepwise model, respectively. Data are presented as odds ratios (95% confidence intervals).

|  | Men (OR, 95% CI) | | Women (OR, 95% CI) | |
| --- | --- | --- | --- | --- |
| Adjusted variables/Predictor variables | Fully Model^*^ | Stepwise Model^*^ | Fully Model^*^ | Stepwise Model^*^ |
| Age | 0.99 (0.99-1) | 0.99 (0.99-1) | 1.03 (1.02-1.04) | 1.03 (1.02-1.03) |
| Urban resident | 0.69 (0.57-0.84) | 0.69 (0.57-0.84) | 0.8 (0.63-1) | 0.79 (0.63-0.98) |
| Smoker | 0.84 (0.69-1.02) | 0.86 (0.71-1.03) | 0.8 (0.47-1.37) | -^#^ |
| Alcohol drinker | 1.03 (0.85-1.25) | -^#^ | 1.27 (0.88-1.84) | -^#^ |
| MHNW | 1 (Reference) | 1 (Reference) | 1 (Reference) | 1 (Reference) |
| MHOW | 1.63 (1.23-2.15) | 1.63 (1.23-2.16) | 1.28 (0.84-1.93) | 1.29 (0.85-1.94) |
| MHO | 1.46 (0.8-2.68) | 1.49 (0.81-2.73) | **1.95 (1.02-3.74)** | **1.99 (1.04-3.82)** |
| MUNW | 1.74 (1.31-2.32) | 1.75 (1.32-2.32) | 3.34 (2.39-4.69) | 3.41 (2.44-4.77) |
| MUOW | 3.57 (2.77-4.6) | 3.61 (2.8-4.66) | 4.45 (3.2-6.2) | 4.55 (3.27-6.32) |
| MUO | 3.47 (2.46-4.91) | 3.53 (2.5-4.98) | 6.31 (4.33-9.18) | 6.56 (4.53-9.51) |
| White Blood Cell | 1.03 (0.98-1.08) | -^#^ | 1.04 (0.99-1.09) | -^#^ |
| TC | 3.2 (2.69-3.8) | 3.21 (2.7-3.81) | 2.92 (2.35-3.64) | 2.98 (2.4-3.7) |
| LDL-C | 0.35 (0.29-0.42) | 0.35 (0.29-0.42) | 0.4 (0.32-0.51) | 0.4 (0.32-0.5) |
| hs-CRP | 1 (0.99-1.01) | -^#^ | 1.02 (1-1.03) | 1.02 (1.01-1.04) |
| Diabetes | 0.71 (0.49-1.03) | 0.72 (0.5-1.05) | 1.08 (0.73-1.61) | -^#^ |

*Abbreviations*: VAI, visceral adiposity index; MHNW, metabolically healthy normal-weight; MHOW, metabolically healthy overweight; MHO, metabolically healthy obese; MUNO, metabolically unhealthy non-obese; MHO, metabolically healthy obese; MUNW, metabolically unhealthy normal-weight; MUOW, metabolically unhealthy overweight; and MUO, metabolically unhealthy obese. TC, total cholesterol; hs-CRP, high-sensitivity C-reactive protein.

^*^Fully model refers to Model 2, and Stepwise model was evaluated from the fully model.

^#^ the adjusted variables/predictor variables excluded from the Stepwise Model.

**Supplementary Figure 1**. Sex-specific prevalence of hyperuricemia according to metabolic health and obesity status (metabolic obesity phenotypes). The metabolic health status was defined by VAI criteria; the obesity status was defined by body mass index. Abbreviations: VAI, visceral adiposity index.


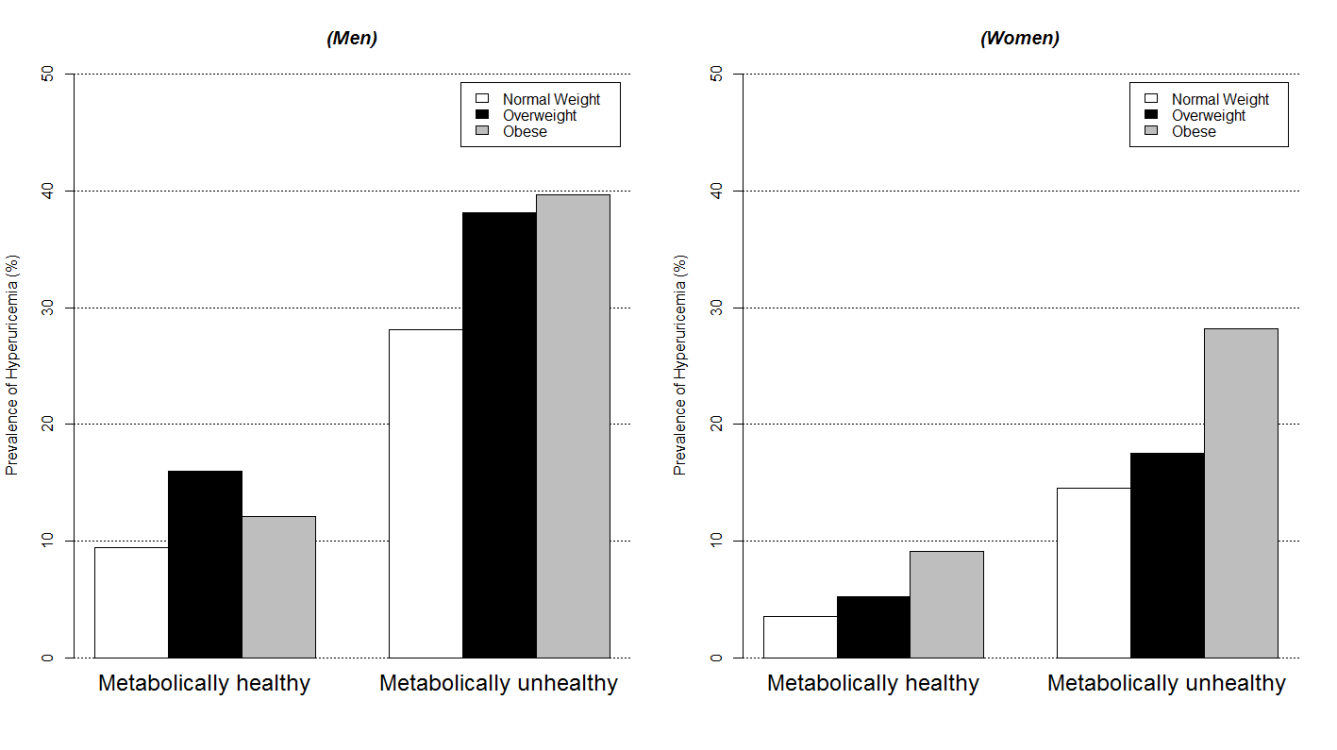


**Supplementary Figure 2**. The DAG underlying the Bayesian network learned from the covariates and hyperuricemia. (a) Averaged DAG with strength of arcs greater than 0.5; (b) Simplified DAG derived from the averaged DAG after retaining arcs with a strength greater than 0.85. The blue dotted lines indicate the edges only existing in the averaged DAG, not in the simplified DAG. Abbreviations: DAG: directed acyclic graph; MetS: metabolic syndrome; TC, total cholesterol; LDL-C, low-density lipoprotein cholesterol; WaistCir: waist circumference.


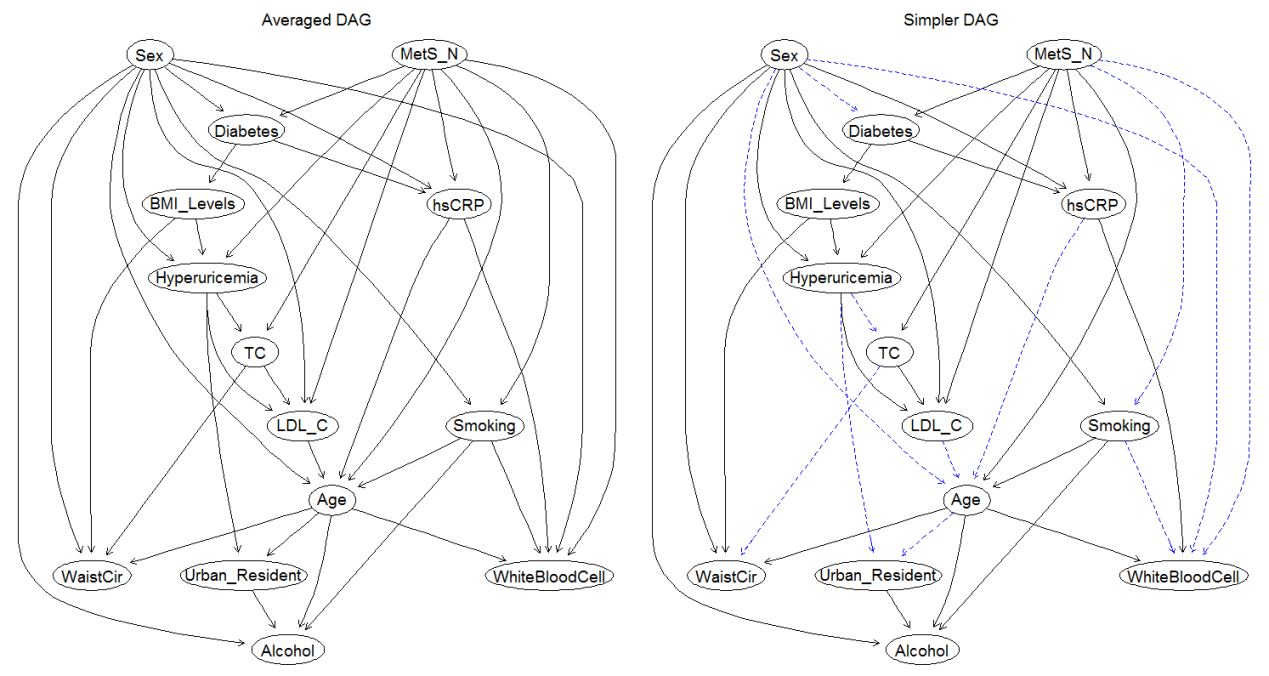

Supplement: Supplementary file 1 [file DataSheet_1.docx]
